# A Deep-Learning-Based Diffusion Tensor Imaging Pathological Auto-Analysis Method for Cervical Spondylotic Myelopathy

**DOI:** 10.3390/bioengineering12080806

**Published:** 2025-07-27

**Authors:** Shuoheng Yang, Junpeng Li, Ningbo Fei, Guangsheng Li, Yong Hu

**Affiliations:** 1 Spinal Division, Orthopedic and Traumatology Center, The Affiliated Hospital of Guangdong Medical University, Zhanjiang 524013, China; shuoheng@connect.hku.hk (S.Y.); junpengli233@gdmu.edu.cn (J.L.); liguangsheng@gdmu.edu.cn (G.L.); 2 Department of Orthopaedics and Traumatology, The University of Hong Kong, Hong Kong, China; u3007507@connect.hku.hk

**Keywords:** cervical spondylotic myelopathy, diffusion tensor imaging, deep learning

## Abstract

Pathological conditions of the spinal cord have been found to be associated with cervical spondylotic myelopathy (CSM). This study aims to explore the feasibility of automatic deep-learning-based classification of the pathological condition of the spinal cord to quantify its severity. A Diffusion Tensor Imaging (DTI)-based spinal cord pathological assessment method was proposed. A multi-dimensional feature fusion model, referred to as DCSANet-MD (DTI-Based CSM Severity Assessment Network-Multi-Dimensional), was developed to extract both 2D and 3D features from DTI slices, incorporating a feature integration mechanism to enhance the representation of spatial information. To evaluate this method, 176 CSM patients with cervical DTI slices and clinical records were collected. The proposed assessment model demonstrated an accuracy of 82% in predicting two categories of severity levels (mild and severe). Furthermore, in a more refined three-category severity classification (mild, moderate, and severe), using a hierarchical classification strategy, the model achieved an accuracy of approximately 68%, which significantly exceeded the baseline performance. In conclusion, these findings highlight the potential of the deep-learning-based method as a decision-making support tool for DTI-based pathological assessments of CSM, offering great value in monitoring disease progression and guiding the intervention strategies.

## 1. Introduction

Cervical spondylotic myelopathy (CSM) is the leading cause of progressive disability in individuals over the age of 65 [1,2]. As spinal cord degeneration is the primary cause of CSM [3,4,5], a precise evaluation of the spinal cord’s pathological condition has become a critical factor in making intervention decisions [6,7]. With the development of neurological medical imaging, this evaluation process often involves its assistance [8,9].

Diffusion Tensor Imaging (DTI) is an advanced magnetic resonance imaging (MRI) technique employed to identify structural abnormalities in neurological tissues [10]. In the pathological analysis of neurological diseases, the diffusion feature metrics derived from DTI, such as Fractional Anisotropy (FA) [11], have proven particularly valuable [12]. Within DTI-based research on CSM, previous studies have demonstrated that diffusive features can reveal pathological changes in CSM patients [13,14,15]. These findings underscore the importance of DTI in understanding CSM’s pathology and guiding clinical interventions.

However, the existing studies on DTI-based pathological analyses have only demonstrated the effectiveness of DTI as a biomarker [12]. These studies have rarely been extended to clinical applications for quantifying CSM’s pathological severity. Specifically, the advancement and broader adoption of current DTI analyses in CSM are constrained by two major challenges.

1. The current analysis process relies heavily on manual methods, which are time-consuming and laborious and require substantial expertise. On the other hand, the analysis process requires regions of interest (ROIs) in the spinal cord to be drawn manually, which is generally believed to be susceptible to human experience and expertise [16,17]. Therefore, the conventional manual approach is inherently limited by subjectivity and variability, resulting in inconsistent outcomes.
2. The selection of spatial features in DTI remains underexplored. In the current research, the ROIs are often manually drawn onto DTI slices that cover the most compressed disc in the spinal cord [9,18]. While this approach has provided valuable insights, it neglects the impact of the compression that occurs in other discs of the spinal cord, which could have a synergistic effect on pathological changes. Consequently, these limitations hinder the scalability of DTI-assisted pathological analyses and restrict their clinical applicability.

In recent years, deep learning has revolutionized the field of medical image/data analysis by enabling automated feature extraction and quantification from high-dimensional data [19,20]. Its ability to process complex, unstructured features with minimal human intervention makes it particularly suitable for addressing the limitations of manual analyses. Despite its success in other applications to studying CSM [21,22], to the best of our knowledge, the feasibility of applying deep learning to DTI-based pathological analyses in CSM still remains under investigation.

The aim of this study is to promote the clinical application of DTI-based pathological analyses of the spinal cord. By designing a deep-learning-based method, this study aims to contribute to the following key aspects:

1. To alleviate the effect of the manual method, the feasibility of deep-learning-based DTI automatic analysis was investigated. The preliminary results demonstrated the effectiveness of an end-to-end pathological analysis, focusing on DTI (the FA values in an image) on both the most compressed spinal disc and the entire spinal cord. This represents the first study to validate the potential of such a deep learning approach in this application field.
2. To investigate the utilization of the spatial information from DTI further, this study designed a multi-dimensional feature fusion mechanism enhanced model, referred to as DCSANet-MD (DTI-Based CSM Severity Assessment Network-Multi-Dimensional). By integrating features from DTI of the maximally compressed cervical disc (2D) and the whole spinal cord (3D), our approach provides an enlarged decision framework for CSM pathological automatic assessment and promotes DTI-assisted clinical management.

## 2. Materials and Methods

### 2.1. Dataset Description

In this study, the experimental data included a total of 176 CSM patients from a single center, including 112 males and 64 females. The ages of the participants ranged from 24 to 98 years, with a mean age of 63.8 ± 13.7 years. All of the participants attended a JOA score assessment [23], with their scores ranging from 2 to 16 and a mean value of 10.5 ± 2.5, as well as cervical DTI and T2-weighted MRI scans. The inclusion criteria required all patients to be clinically diagnosed with CSM without a history of spinal cord surgery. Patients with prior neurological trauma or other neurological disorders were excluded. To ensure data consistency, the time gap in the data collection for DTI, the T2-weighted MRI scans, and the JOA assessments did not exceed three months.

Additionally, it is worth mentioning that while the dataset may exhibit potential imbalances in terms of the gender and age distribution, existing research indicates that the effects of gender- and age-related changes are negligible in DTI analyses of cervical spinal cord disorders [24].

The DTI was acquired using a Philips 3T Achieva scanner (Philips Medical System, Best, The Netherland). For each subject, 12 DTI slices were acquired, covering the entire spinal cord from C2 to C7. For DTI collection, the one-shot Echo Planar Imaging (EPI) sequence was used, and an EPI factor = 35 was used. One b-shell (b values = 600 s/mm^2^) was applied in 15 nonlinear and noncoplanar directions, and the NEX (number of excitations) was equal to 3.

In the pre-processing of the DTI, Spinal Cord Toolbox (Version 2.3) was utilized to derive the DTI diffusion image features [25], including B0 reference extraction and FA map calculation. The FA (Fractional Anisotropy) value map was selected for this study as the target diffusion feature. This selection was intuitively driven by its predominant role in previous research. Subsequently, spatial feature selection for DTI was performed, which included the extraction of the DTI features at the maximally compressed cervical disc (MCCD) of the spinal cord and across the entire spinal cord.

It is worth mentioning that to determine the MCCD DTI slice, this study adhered to the widely recognized criterion based on the smallest anterior–posterior compression ratio [12,26,27]. The calculation process was carried out by experienced researchers, all with clinical backgrounds, using T2-weighted MRI images from the same patient cohort.

**Figure 1:**
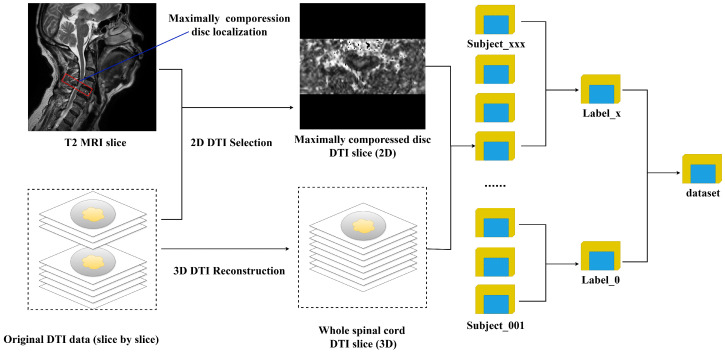
The 2D and 3D DTI identification.

After pre-processing, the selected DTI 2D slices, along with the DTI scans of the entire spinal cord (3D image), were selected as candidate inputs. These two kinds of slices were stored in subject-specific folders labeled with unique patient identifiers. These folders were further organized into severity categories based on the defined labels. As a result, the dataset was structured into a folder tree, as shown in Figure 1.

In terms of the categorization of pathological severity, the JOA score was used as the ground truth to define the severity classes in this study. Specifically, as shown in Table 1, in the binary severity categorization, JOA scores between 0 and 9 were categorized as “severe”, while a score greater than 10 was categorized as “mild”. For the 3-class categorization, while JOA scores ranging from 0 to 9 were still labeled as “severe”, the “mild” category was further divided into two subgroups: JOA scores between 10 and 12 remained as “moderate”, and scores greater than 12 were labeled as “mild”. This definition referenced the related studies [22,28].

### 2.2. The Model Structure

**Figure 2:**
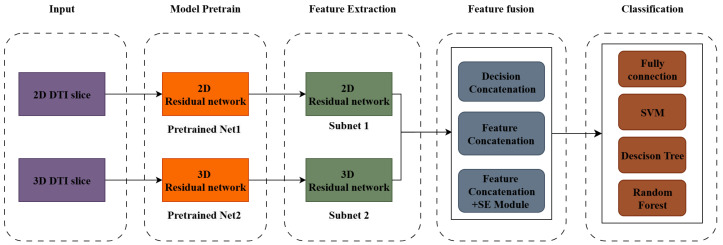
The full structure of DCSANet-MD.

In this study, to explore the feasibility of using a DL-based DTI data analysis to classify the severity of CSM, a multi-dimensional neural network, DCSANet-MD, was proposed. The structure of DCSANet-MD is illustrated in Figure 2. Specifically, the proposed method mainly contains 4 steps: these are model pretraining, multi-dimensional feature extraction, multi-dimensional feature fusion, and classification. In the model pre-training stage, the pre-trained network was acquired from the original single-dimensional network, and the parameters of the pre-trained network were frozen to prevent overfitting. In the subsequent feature extraction stage, 2 pretrained models extracted the features from 2D and 3D DTI data, respectively; then, multi-dimensional information on the condition of the patients’ cervical spines was acquired and fused in a selected mechanism. The final fused features can be processed further using different classifiers, which increases the model’s expandability. The following subsection will introduce the feature extraction step and the feature fusion step in detail.

#### 2.2.1. Feature Extraction

**Figure 3:**
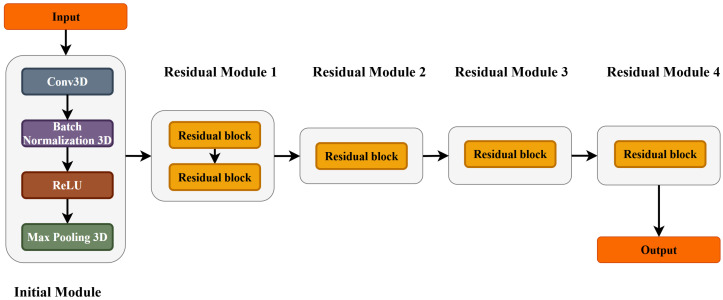
The structure of DCSANet-3D.

The feature extraction stage includes two parallel steps: extracting features from DTI of the MCCD (2D) and of the whole spinal cord (3D). Specifically, residual bottleneck-based 2D and 3D models were proposed. The 2D network named DCSANet-2D was proposed to extract features from the DTI slice of the most compressed cervical spine disc (2D DTI). The proposed network adopts a structure inspired by ResNet18 but simplifies the design by reducing the bottleneck structure, thereby lowering the computational complexity while maintaining high performance. On the other hand, feature extraction for 3D DTI data was conducted using a residual-based 3D network named DCSANet-3D. The structure of DCSANet-3D is an extension of the classical ResNet architecture, where the 2D convolutional and pooling layers are replaced with their 3D counterparts, while preserving the residual connection block design. This adaptation enables the network to capture both spatial and temporal features simultaneously. The details of the DCSANet-3D network architecture are illustrated in Figure 3.

#### 2.2.2. The Feature Integration Mechanism

In addition to feature extraction, the design of the feature integration mechanism is also important in DL for multi-dimensional data to fully leverage the extracted features. In this research, three types of multi-dimensional feature integration mechanisms were proposed: decision fusion, feature fusion, and attention-enhanced feature fusion.

The three proposed mechanisms show potential for classification, respectively.

- Decision fusion is a method that combines the outputs (predictions) of multiple classifiers or models to make a final decision, which simplifies the integration of pre-trained models into an ensemble with minimum further computation, which is beneficial for reducing the model’s potential complexity.
- Feature fusion directly combines the feature representations extracted from different sources of models into a unified feature space; thus, this mechanism is designed to optimize the fusion process, enabling end-to-end learning of the optimal feature representations. After fusion, the combined feature vector is fed into a classifier for the final decision.
- Attention-enhanced feature fusion builds on feature fusion by incorporating attention mechanisms to focus on the most relevant features during the fusion process. In multi-dimensional classification like that in our research, since the multi-source features are noisy and redundant, this mechanism is thought to be especially beneficial. Attention assigns weights to features based on their importance to the classification task, helping the model prioritize critical information while suppressing irrelevant or noisy features, where 2-dimensional modality (2D and 3D) features may have varying importance depending on the pathological context.

Equation (1) shows the formulation of decision fusion, where $D_{1}$ and $D_{2}$ represent the predicted results from 2D and 3D pretrained models, and $D_{C}$ denotes the final merge result.

(1) $$\begin{array}{cc} D_{C} & =Concatenate\left(D_{1},D_{2}\right)=\left[c_{1}+d_{1},c_{2}+d_{2},\ldots ,c_{m}+d_{n}\right]\in R^{p+q} \end{array}$$

The feature fusion mechanism spatially concatenates the original 2-dimensional feature vectors; the formulation is demonstrated in Equation (2), where $F_{1}$ and $F_{2}$ represent the features extracted from the 2D and 3D pretrained models, and $F_{C}$ represents the merged features.

(2) $$\begin{array}{cc} F_{C} & =Concatenate\left(F_{1},F_{2}\right)=\left[a_{1},a_{2},\ldots ,a_{m},b_{1},b_{2},\ldots ,b_{n}\right]\in R^{m+n} \end{array}$$

The attention-enhanced feature fusion mechanism first concatenates the feature vectors and then maps the concatenated vectors into an enhanced representation space by applying the attention mechanism. In this study, the Squeeze−Excitation (SE) block [29] is employed to perform the attention step. In Equation (3), $F_{C}$ represents the concatenated features, and $F_{C}{\_}_{SE}$ denotes the attention-enhanced result.

(3) $$\begin{array}{cc} F_{C}{\_}_{SE} & =Squeeze-Excitation(F_{C}) \end{array}$$

Specifically, to clarify the feature fusion mechanism selection in DCSANet, the feature fusion, decision fusion, and attention-enhanced feature fusion mechanisms were named DCSANet-MD-V1, DCSANet-MD-V2, and DCSANet-MD-V3, respectively. All of the comparison models only applied the feature fusion mechanism.

### 2.3. The Experimental Setting

#### 2.3.1. Data Pre-Processing

For 2D DTI slices, all of the data were transformed from single-channel grayscale images into a three-channel RGB-like form through a channel copy operation. This transformation aimed to accommodate the input requirements of the residual bottleneck. Additionally, a normalization operation was applied to reduce the variance in the loss during training. For 3D DTI data, the original metrics were converted into 3D grayscale image data, and the same normalization strategy was employed. Based on the categorization strategy described above, for both severity categorizations, the dataset was divided into a training set (80%) and a testing set (20%). Five-fold Cross-Validation was performed to obtain the average performance of the proposed model. The initial random seed was generated using the Linux system time to ensure variability in the splits.

#### 2.3.2. Model Training Setting

The hardware used in this experiment included a computer equipped with an Intel i5 CPU (Intel, Santa Clara, CA, USA), 32 GB of RAM (ADATA, Taiwan, China), and an NVIDIA RTX 3080 Ti GPU (Nvida, Santa Clara, CA, USA), which was utilized to accelerate the deep learning training and inference processes. The software environment consisted of the Windows 10 operating system and Visual Studio Code (version 1.102) as the integrated development environment (IDE). The deep learning model was implemented in Python (version 3.8.0) using the PyTorch framework (version 1.10), with the corresponding CUDA version and the cuDNN library applied to enable GPU acceleration.

Table 2 shows the essential hyperparameter selection range and the final best choice in the deep learning experiment. Notably, for models with multi-dimensional (MD) inputs, the parameter freezing strategy was applied, except for the feature fusion and the classifier; this method could thus help to reduce trainable model’s complexity and prevent overfitting.

#### 2.3.3. Comparison Models and the Baseline

To evaluate the predictive potential of the DTI slices from the most compressed section (2D) compared to that of the whole-spine data (3D) for assessing the severity of cervical spondylotic myelopathy (CSM), a total of 12 deep learning (DL) models were utilized and compared with the proposed DCSANet. These models included ResNet18 [30], EfficientNet-B1 [31], SimpleCNN, and their respective 2D, 3D, and multi-dimensional (MD) versions. ResNet18 and EfficientNet-B1 were chosen due to their strong performance, as demonstrated in prior related studies. Conversely, SimpleCNN, an extremely simple CNN network, was incorporated to assess the DL prediction performance under very limited model complexity and computational demands with the current dataset.

In terms of the baseline definition, as this is the first study to test deep-learning-based classification for this application, our experiment used random guessing probabilities as the baseline for all of the proposed and compared models.

#### 2.3.4. Hierarchical Classification (H-Classification)

To address the issue of data scarcity in the 3-class categorization task, the hierarchical classification (H-classification) approach [32] was employed to simplify the learning process and reduce the complexity of classification decision-making. In this experiment, the method restructured the original categorization into a two-step hierarchy based on the criteria outlined in Table 1. The first hierarchical step involves distinguishing between the ‘mild’ and ‘severe’ categories, which is similar to the 2-class categorization task. The second hierarchical step then further divides the ‘mild’ category into ‘mild’ and ‘moderate’ classes.

## 3. Results

### 3.1. Comparison of Different Input Sources

Table 3 presents the results of the models under the two-class category severity categorization setting. The preliminary results indicate that all models achieved a performance exceeding that under random predictions, demonstrating the potential effectiveness of DL-based DTI analysis in predicting CSM’s severity. For the models with 2D inputs, the highest performance was achieved by DCSANet-2D, with an F1 score of 0.7966 and an accuracy of 80.68%. SimpleCNN-2D followed closely, achieving an F1 score of 0.7878 and a slightly higher accuracy of 81.24%. Similarly, for the models with 3D inputs, a comparable performance pattern was observed. The best-performing model was DCSANet-3D, which achieved an F1 score of 0.7371, although its accuracy (76.10%) was marginally lower than that of SimpleCNN-3D (76.13%). Furthermore, models utilizing multi-dimensional (MD) inputs were compared with their counterparts using 2D or 3D inputs, with their superior performance underscoring the advantage of incorporating multi-dimensional data. All of the MD models demonstrated in Table 3 employed feature concatenation as a feature fusion mechanism. Among these, DCSANet-MD-V1 demonstrated the best overall performance, highlighting its structure in this task. On the other hand, among the models that used 2D and 3D inputs, ResNet18 and EfficientNet-B1 exhibited a relatively lower performance in both their accuracy and F1 scores. However, in the MD input mode, EfficientNetB1-MD performed comparably to DCSANet-MD-V1, suggesting its potential for further investigation and comparison.

Table 4 has demonstrated the performance of DCSANet-MD variants, DCSANet-MD-V3 achieved the highest performance, with an accuracy of 82.95% and an F1 score of 0.8135, demonstrating the best classification performance compared with all of the models using two-category severity classification. This result shows the effectiveness of the V3 architecture in optimizing the feature fusion outcomes. In terms of DCSANet-MD with classifier variants, when the classifier in DCSANet-MD-V3 was replaced with a machine learning classifier, its performance generally declined compared to that of the fully connected network. On the other hand, all three models with machine learning classifiers show a similarly fair performance in both their accuracy and F1 scores, which is a fair result compared with those for DCSANet-MD-V1 and DCSANet-MD-V2.

### 3.2. The Effectiveness of DCSANet in Predicting the Three-Class Severity Categories

To evaluate the models’ performance in a more refined three-class categorization task, a hierarchical classification strategy was employed by DCSANet to address the issue of data scarcity, as the number of samples in each severity class was limited in this setting. Table 5 presents the results of both the standard three-class classification and the hierarchical classification strategy used to differentiate between the ‘mild’ and ‘moderate’ categories. These experiments were conducted using 2D, 3D, and MD input methods.

It can be observed that the accuracy of the original classification exceeds that of random selection, demonstrating the effectiveness of deep learning methods in this task. Notably, the H-classification strategy consistently outperforms the standard three-class classification strategy (with the only exception being the SimpleCNN-2D model), further highlighting the effectiveness of the hierarchical approach in improving the classification performance under challenging conditions.

**Figure 4:**
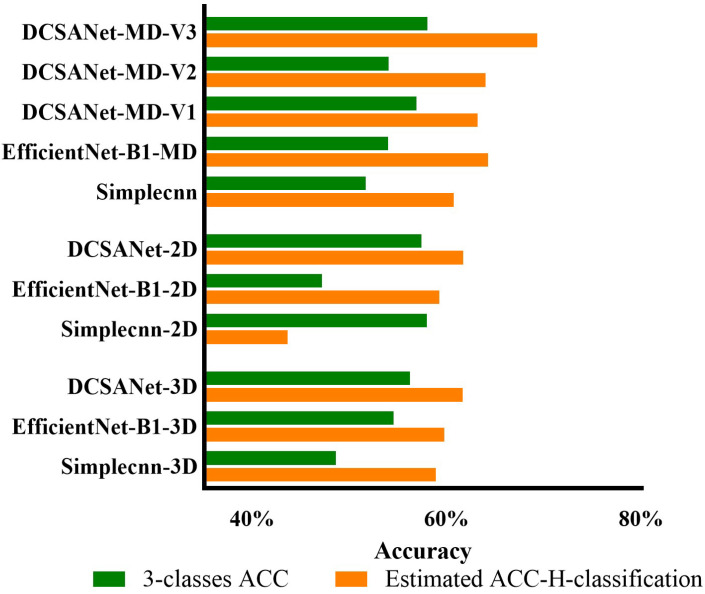
Comparison of hierarchical classification with vanilla classification strategy.

To demonstrate the effectiveness of the hierarchical approach in improving the classification performance under data-constrained conditions, Figure 4 presents the performance gap between the two classification strategies. DCSANet-MD-V3 achieves the highest performance in both strategies, with the ACC estimated using the H-classification strategy exceeding 70% and an accuracy for standard three-class classification close to 60%.

## 4. Discussion

The aim of this study is to promote the clinical application of DTI to pathological analyses of the spinal cord. To achieve this objective, this research contributes two critical points: the application of a deep learning (DL)-based DTI analysis as an alternative to the manual method for assessing CSM’s pathological severity and the utilization of multi-dimensional integration to leverage the spatial features of DTI more effectively. In terms of the first aspect, several DL models were evaluated using two types of DTI inputs: the most compressed cervical disc (the 2D form) and data from the entire spinal cord (the 3D form). While DTI images have previously been utilized for CSM analyses, most existing studies have relied on manual analysis methods [18,33]. In contrast, this study implemented an end-to-end automatic analysis using DL technology, which significantly reduced the impact of manual intervention and enhanced the analysis’s efficiency. Moreover, the previous manual methods have not been performed under an increasing sample size, where requires greater time consumption. Conversely, the sample size in our study is considered one of the largest datasets of cervical DTI samples (176 subjects) according to the known literature [14,16], which also highlights the advantage of the DL-based automatic method in analysis practice. In terms of the second aspect, the impact of different cervical discs demonstrates the potential to leverage spatial information from DTI further. To investigate potential performance improvements, models incorporating multi-dimensional DTI inputs were compared. To the best of our knowledge, this investigation was the first attempt to integrate the spatial features of DTI for evaluations of the pathological condition in CSM, which increased the representation search space for such analyses. To enhance the flexibility of utilizing spatial features from DTI further, different feature fusion mechanisms and classifiers were also investigated. Ultimately, an attention-based feature fusion mechanism was employed to improve the adaptability of the spatial feature fusion process. Additionally, this study extended the severity classification task to a three-class severity categorization. A hierarchical classification strategy was introduced to optimize the data utilization. This additional attempt therefore aimed to promote the clinical application of DTI in precision medicine.

### 4.1. The Effectiveness of Deep-Learning-Based Pathological Severity Predictions Using DTI in CSM

In terms of the feasibility of applying deep learning to automatic predictions of pathological severity using DTI in CSM, based on the acquired results, since all of the presented models exceeded the results of random classification, the potential of the deep-learning-based methods and their feasibility have been validated. On the other hand, the results also demonstrated the contribution of different parts of the spinal cord to CSM’s severity, including the most compressed section (2D) and the entire spinal cord (3D). Obviously, all damage to the spinal cord could affect CSM’s pathology, rather than just the most compressed disc, and the varying performance between the 2D and 3D DTI slices indicates the potential of applying combined data dimensions. Therefore, an MD model was proposed to explore the value of the joint contribution of multi-dimensional DTI. The results shown in Table 3 and Table 4 indicate that an MD-data-based analysis consistently outperforms solely applying a 2D or 3D analysis across all model architectures. This finding demonstrates the advantage of incremental information acquired from data combination.

### 4.2. Analysis of the Model Performance in Two-Class Categorization

To achieve the aim of automatic analyses of DTI in CSM, this study specially proposes the DCSANet model zoo (including 2D, 3D, and MD versions) to improve the feature extraction. In terms of the performance evaluation in two-class categorization, the DCSANet model zoo consistently achieved the highest or near-highest performance across all input source settings, showcasing its robustness and suitability for this task. On the other hand, the SimpleCNN model zoo follows closely in its performance, particularly in the 2D input setting, where it achieves the highest accuracy. This suggests that this architecture is well suited to small sample sizes, though it slightly lags behind DCSANet in terms of its F1 scores. Models with an EfficientNet-B1 structure, while underperforming with 2D and 3D inputs, demonstrate significant improvements in the MD setting, and the results achieved are comparable to those of the top-performing models. This suggests that EfficientNet-B1-MD also effectively leverages multi-dimensional data, but its unstable performance makes it a controversial candidate for such an analysis task. ResNet18 exhibits the lowest performance across all input sources, despite its wide application to medical image analyses [34]. This finding demonstrates that it may not be as well suited to the DTI modality and the limited size of the current datasets. Consequently, the DCSANet-MD-V1 models are considered the most suitable model because of their feature extraction ability.

### 4.3. The Effectiveness of the Feature Fusion Mechanism

To evaluate the effectiveness of different feature fusion mechanisms further, the DCSANet-MD-V1 model was selected as a base model to compare with the decision fusion mechanism and the attention-enhanced fusion mechanism (named DCSANet-MD-V2 and DCSANet-MD-V3, respectively). According to Table 4, DCSANet-MD-V3 clearly outperforms DCSANet-MD-V1 and DCSANet-MD-V2 in both its accuracy and F1 score. This result suggests that the attention-enhanced feature fusion mechanism introduced into DCSANet-MD-V3 is more effective for processing multi-dimensional features. The SE attention module applied in DCSANet-MD-V3 is generally believed to enhance the model’s ability by reweighting the extracted feature maps without explicitly increasing the model’s complexity [29]. These improvements can be inferred according to the enhanced representational capacity due to the SE module in DCSANet-MD-V3. Conversely, the similar performance observed between DCSANet-MD-V1 and DCSANet-MD-V2 indicates the similar effectiveness of these two feature fusion mechanisms, with only minor differences influencing the balance between the accuracy and F1 score due to the slightly higher F1 score achieved by DCSANet-MD-V1.

### 4.4. The Effectiveness of the Machine Learning Classifier

Besides feature extraction, this study also explored the effectiveness of different classifier variants. According to Table 4, both the fully connected (FC) classifier originally applied in DCSANet-MD-V3 and other traditional classifiers, including SVM, Random Forest (RF), and a Decision Tree (DT), are demonstrated. The results showed that these classifiers experienced a performance decline: the SVM showed the largest drop in its F1 score (72.38%) and accuracy (76.71%), indicating that it was less effective in leveraging the complex feature representations extracted by DCSANet-MD-V3. RF performs slightly better than the SVM, but its overall performance is still modest. Interestingly, the DT achieves the lowest accuracy (75.03%) but a relatively better F1 score (75.75%) compared to the SVM and RF. This suggests that the DT classifier may be good at balancing the predictions in each class. The overall comparison results highlight the representation flexibility of the pure deep neural network model (including the FC part) in terms of the utility of the fused features, which also coincides with its advantages in DL research [35].

### 4.5. Analysis of the Model Performance in Three-Class Categorization

To provide a more comprehensive investigation, a three-class category prediction task was designed to evaluate the robustness of the proposed DCSANet model. Similarly to the two-class category, the result shows that multi-dimensional input (MD) provides significant performance gains for all models. Obviously, the ability to integrate complementary information from 2D and 3D inputs into MD models can be concluded as critical to improving the predictions. The DCSANet models demonstrate a superior performance across all input modalities again, with DCSANet-MD-V3 achieving the best results for three-class classification. This experiment demonstrates the robustness of the DCSANet architecture for handling different severity category tasks. To improve the classification performance further, the effectiveness of the hierarchical classification (H-classification) strategy was explored in three-class categorization. The results in Table 5 and Figure 4 demonstrate that the H-classification strategy achieves a higher accuracy than that of the direct classification strategy across most models. This result is consistent with our expectations, as hierarchical classification simplifies the decision-making process by dividing it into multiple levels and leveraging the hierarchical relationships between classes. Notably, the performance gap between the two strategies is particularly noticeable in EfficientNet-B1, where the model exhibits significant difficulty handling the complexity of three-class classification, which may indicate the same unstable performance mentioned in the binary categorization task.

### 4.6. Analysis-Associated Factors May Interfere with DTI Assessments

Besides the models’ performance varying in different settings, there are also some common factors in all of the proposed methods that may affect DTI-based pathological analyses.

1. The Segmentation Process for DTI: In this study, the entire DTI slice was used as a feature matrix to identify CSM’s severity. Although this design aims to realize the primary objective of providing an end-to-end method, in manual methods, a segmentation process is typically applied to extract anatomical-level structures from the background [33]. The absence of segmentation in our approach may have affected the analysis and the interpretation of DTI features at the anatomical structure level.
2. Unexplored Relationships Between Multiple Compressed Discs: As described in Section 1, cervical compression frequently affects multiple discs. However, due to manpower and time constraints, most existing studies have focused only on the maximally compressed cervical disc (MCCD) or the entire cervical spinal cord [14,16]. While our method innovatively integrates both into the analysis, the relationship among the number and location of affected cervical discs (rather than the entire spinal cord) and the severity of CSM has not been thoroughly investigated. This limitation may have impacted the precision of the model’s performance.
3. Selection of DTI Diffusive Features: While DTI provides several diffusive features, besides the FA value, other features such as the Mean Diffusivity (MD), Axial Diffusivity (AD), and Radial Diffusivity (RD) can also be used for analysis [36]. This study primarily utilized FA as the target feature because it is the most commonly used index and represents a combination of the other three metrics. However, the relationships among MD, AD, and RD in CSM analysis remain unclear. A more comprehensive study is needed to explore these relationships and develop synthesis methods for integrating these indices into the analysis.

### 4.7. Clinical Relevance and Research Value

The aim of the proposed methods is to facilitate the clinical application of DTI; therefore, the clinical relevance and research value of this work are particularly significant.

In terms of its clinical relevance, the current assessment of CSM’s pathological severity largely depends on symptom-based rating methods, with the Japanese Orthopaedic Association (JOA) score and its modified version (mJOA) being the most widely used [23]. However, these indices often exhibit a mismatch with the extent of pathological damage, as they fail to comprehensively capture the underlying neurological tissue damage [26,37]. Diffusion Tensor Imaging (DTI) is a promising imaging modality with the potential to reveal pathological damage at the neurological tissue level.

The objective of this study is to investigate the feasibility of leveraging DTI data to quantify the severity of CSM. By developing an end-to-end DTI analysis framework specifically designed for severity quantification, this study further evaluates the potential of applying DTI data as a clinical index in CSM management. Additionally, this automated approach eliminates the need for manual feature selection in DTI analysis, thereby simplifying the workflow and enhancing the accessibility of DTI data for routine clinical practice.

In terms of the research value, the findings of this study proved the potential of the proposed deep-learning-based DTI analysis for pathological severity assessments in CSM. By providing automatic quantification, this method could assist clinicians in monitoring CSM’s progression and proposing treatment strategies with the minimum subjectivity. Furthermore, the model’s ability to analyze multi-dimensional DTI slices could complement the existing clinical assessments, offering a more reliable approach to evaluating the progression of spinal cord damage.

### 4.8. Limitations and Future Direction

#### 4.8.1. Limitations

Despite its promising results, this study has several limitations that should be acknowledged.

First, the dataset used in this study consists of 176 CSM patients from a single center. While this represents one of the largest sample sizes reported in existing studies [14,16], it is only sufficient for proof-of-concept research. For broader clinical applications, the development of larger and more diverse multi-center datasets is essential. We believe that utilizing large datasets would enable better validation of the model’s performance across varied patient populations, ultimately improving its clinical reliability.

Second, although a hierarchical classification strategy was employed, the prediction performance for three-class severity categorization remained relatively modest. This indicates a need for further refinement of the model to enhance its discriminatory ability, particularly in capturing features between severity levels.

Third, the interpretability of the proposed method requires further improvement. The challenge of interpretability in deep learning models is a widely debated topic in AI and interdisciplinary research [38,39]. Improving the interpretability of the model would not only align with the clinical ethics mandate for transparency in decision-making but also provide valuable insights for the development of future methodologies.

Finally, this study exclusively explored the application of the model to DTI slices, which may have resulted in the exclusion of certain special cases that are less sensitive to this data modality. Future research should consider incorporating additional imaging modalities or clinical data to ensure a more comprehensive assessment of CSM’s pathology and its diverse presentations.

#### 4.8.2. Future Directions

To address the aforementioned limitations, several directions have been proposed.

Firstly, to mitigate the challenges posed by a limited dataset size, future research should prioritize expanding the dataset to include a larger number of patients from diverse backgrounds and clinical settings. In particular, constructing external datasets would enhance the generalization capability of the deep learning models and reduce the risk of overfitting associated with relatively small datasets.

Secondly, to improve the prediction accuracy in more detailed classification tasks, the feasibility of utilizing advanced model architectures should be investigated. For instance, transformer-based networks, which have been widely adopted in AI and interdisciplinary research, show great potential [40,41]. The application of such cutting-edge models could enhance feature extraction and interpretation further in DTI analysis, particularly when the issue of data scarcity is alleviated.

Thirdly, to address the interpretability challenges with deep learning models, two potential directions should be explored. On the one hand, although a standardized evaluation method for interpretability has yet to be established, some progress has been made in the interpretability of deep-learning-based AI systems. Extending these studies into AI-assisted medical research could provide valuable insights, and an analysis framework that can fit the clinical research (limited datasets) is expected [42,43]. On the other hand, incorporating domain knowledge into AI-assisted medical research presents a more defined pathway [44]. In the model design stage of our study, inspired by expert knowledge of CSM pathology, we implemented a sub-network structure to analyze both the maximally compressed spinal disc and the entire spinal cord. However, achieving more robust interpretability will require a deeper understanding of the pathological mechanisms of the spinal cord and their impact on CSM progression. Advancements in clinical research are thus essential to support this effort.

Finally, to address the potential limitations of DTI in certain CSM cases, we propose the integration of multi-modal analysis [45], to explore its feasibility in enhancing the model’s performance. While the addition of data modalities may increase the complexity of severity predictions’ interpretability, combining multi-modality data could expand the decision-making space and provide a more holistic assessment of CSM’s pathological severity. For example, T2-weighted MRI and clinical records have already been proven to be effective tools for CSM severity assessments [23,46,47]. Developing multi-modal algorithms that integrate these modalities with DTI data could offer a more comprehensive representation of CSM’s pathology.

## 5. Conclusions

The proposed DTI-based multi-dimensional image fusion model demonstrates significant potential to automate the assessment of the pathological severity of the spinal cord in CSM. The integration of the proposed method into clinical workflows could provide valuable support for monitoring CSM’s progression and supporting intervention decision-making.

## Figures and Tables

**Table 1:** The categorization criteria definitions.

Two-Class Categorization	Two-Class Severity Level	Three-Class Categorization	Three-Class Severity Level
JOA≤9	Severe	JOA≤9	Severe
JOA≥10	Mild	JOA=[10−12]	Moderate
N/A	N/A	JOA≥13	Mild

N/A is an abbreviation of ‘Not applicable’.

**Table 2:** The training hyperparameter settings.

Hyperparameter Item	Parameter Space	Choice
Optimizer	[Adam, SGD]	Adam
Learning rate	[1 × 10^−4^, 3 × 10^−4^, 1 × 10^−3^, 3 × 10^−3^]	1 × 10^−4^
Batch size	[8, 16, 32]	8
Training epoch	[30, 50, 100]	50
Loss	[Focal loss, CE loss]	Focal loss
Classification rebalance weight-2 classes	[1:1, 1:3]	1:3
Classification rebalance weight-3 classes	[1:2:1, 1:1:1]	1:2:1

**Table 3:** A performance comparison of the models under 2-class categorization.

Input Source	Model Name	ACC 2-Class Categorization	F1 2-Class Categorization
2D	resnet-18-2D	71.59%	0.6997
	EfficientNet-B1-2D	56.73%	0.5883
	Simplecnn-2D	81.24%	0.7878
	DCSANet-2D	80.68%	0.7966
3D	resnet-18-3D	68.17%	0.6696
	EfficientNet-B1-3D	74.98%	0.7330
	Simplecnn-3D	76.13%	0.7259
	DCSANet-2D	76.10%	0.7371
2D-3D	resnet-18-MD	72.70%	0.7231
	EfficientNet-B1-MD	78.43%	0.7756
	Simplecnn-MD	78.41%	0.7643
	DCSANet-MD-V1	79.54%	0.7771

**Table 4:** The performance of DCSANet with different feature fusion mechanisms and classifiers under 2-class categorization.

Model	ACC 2-Class Categorization	F1 2-Class Categorization
DCSANet-MD-V1	79.54 %	0.7771
DCSANet-MD-V2	79.54 %	0.7724
DCSANet-MD-V3	82.95 %	0.8135
DCSANet-MD-V3-SVM	76.71 %	0.7238
DCSANet-MD-V3-RF	77.27 %	0.7372
DCSANet-MD-V3-DT	75.03 %	0.7575

**Table 5:** The performance of the models under 3-category classification (including 2nd-level H-classification and direct 3-class classification).

Input	Model	H-CLASS-2nd Level		3-CLASS	
		**ACC**	**F1**	**ACC**	**F**1
2D	Simplecnn-2D	65.30%	57.34%	57.94%	50.58%
	EfficientNet-B1-2D	59.31%	59.37%	47.17%	45.99%
	DCSANet-2D	64.62%	53.74%	57.37%	52.67%
3D	Simplecnn-3D	67.70%	58.61%	48.57%	47.99%
	EfficientNet-B1-3D	67.64%	57.68%	54.52%	50.68%
	DCSANet-3D	71.50%	67.19%	56.22%	49.58%
2D-3D	Simplecnn-MD	66.13%	60.85%	51.67%	45.63%
	EfficientNet-B1-MD	72.89%	71.33%	53.97%	51.18%
	DCSANet-MD-V1	69.12%	63.03%	56.84%	52.16%
	DCSANet-MD-V2	70.60%	64.37%	53.98%	46.17%
	DCSANet-MD-V3	75.29%	71.86%	57.98%	54.81%

ACC is is an abbreviation of ‘Accuracy Index’.

## Data Availability

The data presented in this study are available on request from the corresponding author.

## Abbreviations

The following abbreviations are used in this manuscript:

CSM	cervical spondylotic myelopathy
DTI	Diffusion Tensor Imaging
FA	Fractional Anisotropy
DL	deep learning
DCSANet	DTI-Based CSM Severity Assessment Network
MCCD	maximally compressed cervical disc
